# *Notes from the Field*: Cannabis Detection Among Overdose Deaths — United States, 2021–2025

**DOI:** 10.15585/mmwr.mm7534a2

**Published:** 2026-09-03

**Authors:** Lauren J. Tanz, Zerleen S. Quader, Christine L. Mattson, Alana M. Vivolo-Kantor

**Affiliations:** 1 Division of Overdose Prevention, National Center for Injury Prevention and Control, CDC.

**Summary**

**What is already known about this topic?**

In the United States, cannabis is the most commonly used drug that is illegal under federal law; however, little is known about the frequency of cannabis detection in drug overdose deaths or age distribution of persons who died of an overdose with cannabis detected.

**What is added by this report?**

During January 2021–June 2025, cannabis was detected in 21.0% of overdose deaths; the percentage was stable over time. Cannabis was listed as causing death in 0.8% of overdose deaths. Percentages of deaths with cannabis detected were highest among adolescents aged 12–17 years and decreased with increasing age.

**What are the implications for public health practice?**

Community-based approaches to preventing and reducing youth cannabis use have the potential to improve the health of youths now and as they move into adulthood.

In the United States, cannabis is the most commonly used drug that is illegal under federal law*; in 2024, an estimated 64.2 million persons aged ≥12 years used cannabis (*1*). Frequent cannabis use is associated with various adverse health effects, including memory impairment, cannabis hyperemesis syndrome, chronic psychosis disorders, and cannabis use disorder (i.e., cannabis use leading to clinically significant impairment or distress) (*2*,*3*). Although cannabis alone rarely causes fatal overdoses (*2*), detection on postmortem toxicology testing among overdose deaths might help identify populations at high risk for other adverse health consequences (e.g., substance use disorders), including death, and help guide tailored prevention efforts. It is not known how often cannabis is detected among overdose deaths and whether detection varies by age.

## Investigation and Outcomes

### Data Source

CDC’s State Unintentional Drug Overdose Reporting System (SUDORS)^†^ collects data on unintentional and undetermined intent drug overdose deaths from death certificates, medical examiner and coroner reports, and postmortem toxicology reports. SUDORS captures all drugs detected by postmortem toxicology, even those not ruled by a medical examiner or coroner to have been involved in (i.e., caused) the death. Percentages of overdose deaths with cannabis detected were calculated by age and year for deaths that occurred during January 2021–June 2025 in 31 states and the District of Columbia.^§^ Cannabis included cannabinoids derived from cannabis (e.g., cannabidiol, tetrahydrocannabinol [THC], and tetrahydrocannabivarin) and dronabinol and excluded synthetic cannabinoids (synthetic substances not derived from cannabis).^¶^^,^** This activity was reviewed by CDC, deemed not research, and conducted consistent with applicable federal law and CDC policy.^††^

### Overdose Deaths with Cannabis Detected

Among 209,166 overdose deaths during January 2021–June 2025, cannabis was detected in 43,880 (21.0%); the percentage of overdose deaths with cannabis detected was stable over time (Figure). Cannabis was listed as causing death (i.e., involved) in 0.8% (1,687) of all overdose deaths and was the only drug involved in 0.004% (nine). The percentage of deaths with cannabis detected decreased with increasing decedent age. Among decedents aged 12–17 years, cannabis was detected in 42.7% (439) of overdose deaths. Within this age group, the percentage decreased from 49.2% in 2021 to 35.9% in 2024 and increased to 44.6% during January–June 2025. The percentage of deaths with cannabis detected was lowest among adults aged ≥50 years (13,076; 16.7%); however, the percentage increased slightly from 14.8% in 2021 to 18.1% during January–June 2025. Among all deaths with cannabis detected, most involved illegally manufactured fentanyls (73.5%) or stimulants (e.g., cocaine or methamphetamine; 60.4%), including 88.6% and 69.0%, respectively, of deaths among adolescents aged 12–17 years.

**FIGURE:**
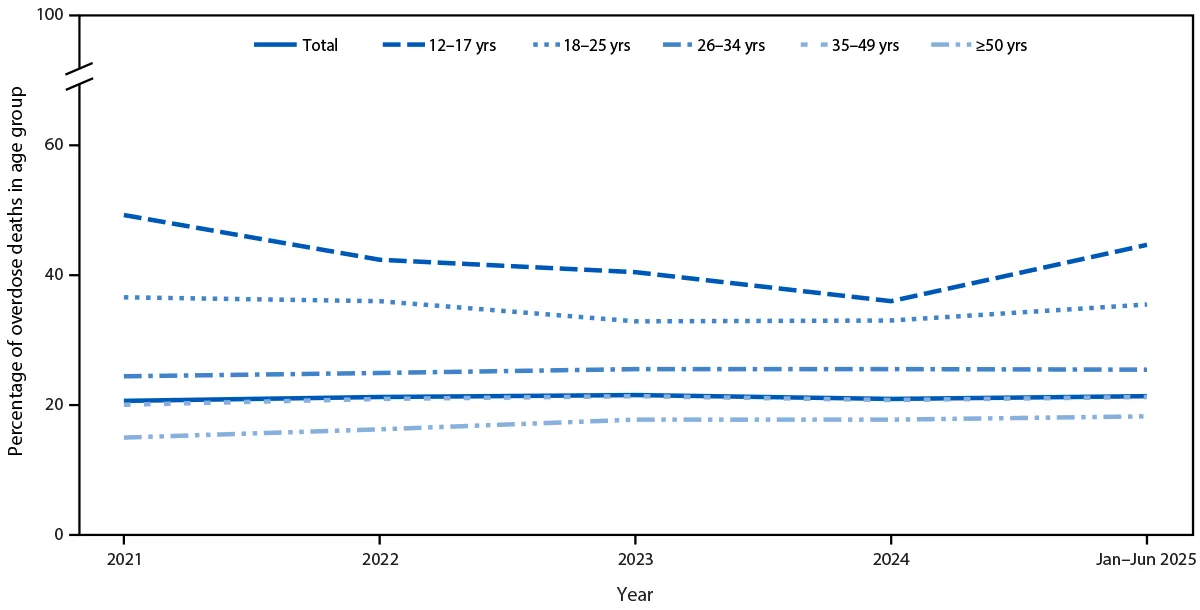
Percentage* of overdose deaths with cannabis detected, by decedent age group — State Unintentional Drug Overdose Reporting System, United States,^†^ January 2021–June 2025 * Percentages were calculated among overdose deaths that occurred in the specified age group. During 2021–June 2025, 1,028 overdose deaths occurred among decedents aged 12–17 years, 13,014 among those aged 18–25 years, 39,842 among those aged 26–34 years, 76,807 among those aged 35–49 years, and 78,475 among those aged ≥50 years. Total sample size was 209,166 deaths. ^†^ Alaska, Arizona, Arkansas, Colorado, Connecticut, Delaware, District of Columbia, Georgia, Hawaii, Illinois, Iowa, Kansas, Kentucky, Louisiana, Maine, Maryland, Minnesota, Nebraska, Nevada, New Jersey, New Mexico, North Carolina, Ohio, Oklahoma, Oregon, Pennsylvania, Rhode Island, Utah, Vermont, Virginia, Washington, and West Virginia. For inclusion, jurisdictions were required to report ≥75% of overdose deaths in their jurisdiction and have toxicology reports for ≥75% of deaths for the full period of analysis. Analyses were restricted to deaths with toxicology reports (95.2% of deaths in included jurisdictions).

## Preliminary Conclusions and Actions

One in five overdose deaths during January 2021–June 2025 had cannabis detected; the percentage was highest among adolescents aged 12–17 years, despite this age group having the lowest prevalence of self-reported past-year cannabis use in the general population and stable or slightly declining prevalence of cannabis use in recent years (*1**,**4*). Although past-year prevalence of self-reported cannabis use increased during 2021–2024 among adults aged ≥26 years (*1*), detection in overdose deaths remained relatively stable. Fewer than 1% of deaths had cannabis listed as a cause of death, and all but nine deaths had at least one other drug involved. This evidence indicates that overdose deaths caused by cannabis are rare, and detection of cannabis among overdose deaths is primarily indicative of polysubstance use. Because cannabinoids are rarely implicated in the cause of death (*2*), cannabinoid screening and confirmatory testing is not universally conducted. Thus, the identification of cannabis use among overdose deaths is likely underestimated.

Cannabis use among youths represents an important opportunity for early intervention to prevent cannabis use disorder and address other substance use disorders and related adverse health effects (*5*). Implementation of programs, such as CDC’s Drug Free Communities Support Program and Evidence-Based Strategies to Prevent Youth Substance Use (ENGAGE), that use interdisciplinary community-based approaches to prevent and reduce youth substance use, including cannabis use, might improve the health of youths in the United States now and, over time, reduce substance use in adults.

## Footnotes

* Although cannabis is illegal under federal law, 41 states and the District of Columbia allow medical use of cannabis, and 24 states and the District of Columbia allow or regulate nonmedical use of cannabis by adults. State Medical Cannabis Laws | National Conference of State Legislatures

† For SUDORS, overdose deaths were identified by funded jurisdictions both through *International Classification of Diseases, Tenth Revision *underlying cause-of-death codes X40–44 (unintentional) and Y10–14 (undetermined intent) and through text searches of literal cause-of-death fields on death certificates that indicated acute drug toxicity.

§ Alaska, Arizona, Arkansas, Colorado, Connecticut, Delaware, District of Columbia, Georgia, Hawaii, Illinois, Iowa, Kansas, Kentucky, Louisiana, Maine, Maryland, Minnesota, Nebraska, Nevada, New Jersey, New Mexico, North Carolina, Ohio, Oklahoma, Oregon, Pennsylvania, Rhode Island, Utah, Vermont, Virginia, Washington, and West Virginia. For inclusion, jurisdictions were required to report ≥75% of overdose deaths in their jurisdiction and have toxicology reports for ≥75% of deaths for the full period of analysis. Analyses were restricted to deaths with toxicology reports (95.2% of deaths in included jurisdictions).

¶ Screening for cannabinoids in postmortem fluids typically uses immunoassay analysis that cannot distinguish delta-8-THC and delta-9-THC. Definitive identification of delta-8-THC, delta-9-THC, and their metabolic byproducts necessitates confirmatory testing.

** Among deaths in this analysis with cannabis detected, delta-9-THC was detected in 73.1%, delta-8-THC in 1.1%, dronabinol in none, and a different cannabis derivative (e.g., cannabidiol, cannabigerol, or tetrahydrocannabivarin) in 0.1%; cannabis that was not otherwise specified as a specific cannabinoid detected (e.g., marijuana metabolite) was detected in 26.6%. Categories are not mutually exclusive except for the category of cannabis not otherwise specified as a specific cannabinoid.

†† 45 C.F.R. part 46.102(l)(2), 21 C.F.R. part 56; 42 U.S.C. Sect. 241(d); 5 U.S.C. Sect. 552a; 44 U.S.C. Sect. 3501 et seq.
